# Development and Alpha Testing of QuitIT: An Interactive Video Game to Enhance Skills for Coping With Smoking Urges

**DOI:** 10.2196/resprot.2416

**Published:** 2013-09-11

**Authors:** Paul Krebs, Jack E Burkhalter, Bert Snow, Jeff Fiske, Jamie S Ostroff

**Affiliations:** ^1^New York University School of MedicineDepartment of Population HealthNew York, NYUnited States; ^2^Memorial Sloan-Kettering Cancer CenterDepartment of Psychiatry and Behavioral SciencesNew York, NYUnited States; ^3^Muzzy Lane SoftwareNewburyport, MAUnited States

**Keywords:** smoking cessation, health promotion game, tobacco, quitting self efficacy, behavioral medicine, virtual reality

## Abstract

**Background:**

Despite many efforts at developing relapse prevention interventions, most smokers relapse to tobacco use within a few months after quitting. Interactive games offer a novel strategy for helping people develop the skills required for successful tobacco cessation.

**Objective:**

The objective of our study was to develop a video game that enables smokers to practice strategies for coping with smoking urges and maintaining smoking abstinence. Our team of game designers and clinical psychologists are creating a video game that integrates the principles of smoking behavior change and relapse prevention. We have reported the results of expert and end-user feedback on an alpha version of the game.

**Methods:**

The alpha version of the game consisted of a smoking cue scenario often encountered by smokers. We recruited 5 experts in tobacco cessation research and 20 current and former smokers, who each played through the scenario. Mixed methods were used to gather feedback on the relevance of cessation content and usability of the game modality.

**Results:**

End-users rated the interface from 3.0 to 4.6/5 in terms of ease of use and from 2.9 to 4.1/5 in terms of helpfulness of cessation content. Qualitative themes showed several user suggestions for improving the user interface, pacing, and diversity of the game characters. In addition, the users confirmed a high degree of game immersion, identification with the characters and situations, and appreciation for the multiple opportunities to practice coping strategies.

**Conclusions:**

This study highlights the procedures for translating behavioral principles into a game dynamic and shows that our prototype has a strong potential for engaging smokers. A video game modality exemplifies problem-based learning strategies for tobacco cessation and is an innovative step in behavioral management of tobacco use.

## Introduction

### Background

Approximately 40-50% of smokers relapse to tobacco use even after a sustained period of abstinence [1-3]. Such high rates of relapse to smoking have stimulated extensive research and theory development directed toward prevention of relapse [4-7]. The effectiveness of relapse prevention interventions, however, has been mixed. A recent systematic review of studies on prevention of smoking relapse showed minimal efficacy for advice, written materials, mailings, and telephone contact [4]. However, the review showed that evidence exists for interventions based on behavioral rehearsal to help smokers identify, model, and practice coping strategies to identify and manage smoking cues. Treatment strategies based on these learning principles promote skill acquisition, bolster confidence in coping with smoking cues, and reduce relapse to smoking [8]. A computer game enables individuals to interact with realistic environments and thus is uniquely suited for engaging smokers in skill-building activities for managing urges to smoke. Games and simulations can serve as powerful tools because they encompass many aspects of human learning such as engagement, problem-solving, receiving corrective feedback, and repetition [9-11]. In addition, games are increasingly popular across a wide population. For instance, as of 2011, 47% of game players were women and 37% were over the age of 36 [12]. A recent systematic review of video games for promoting health outcomes showed that video games offer significant benefit for psychological therapy, health education, and disease management [13].

### Status of Cessation Games

Few e-games have been specifically created for promoting smoking cessation in adults. Of those that focus on smoking, one (Rex Ronan) is a game for prevention of smoking in adolescents [14] and another (Blast n Quit) is an arcade-type game in which the users shoot enemies that could compromise their quit attempts. In addition, mobile game apps have been developed, such as Nicot, which allows users to crush virtual cigarettes, and Lit 2 Quit, which guides users through breathing exercises. However, most apps primarily provide assistance in cigarette tracking [15]. None of these games or apps is deeply grounded in applying behavioral change theory to tobacco cessation. Effective tobacco cessation and relapse prevention interventions require a range of complex strategies such as identifying tobacco use triggers, engaging in new substitute coping behaviors, seeking social support from family and friends about tobacco use, modifying one’s internal dialogue, and dealing with inevitable slips [6].

Translating these evidence-based strategies into a game calls for an interactive, immersive application in which the user can learn and practice these techniques in a realistic context. Through repeated exposure to conditioned cues to smoke (eg, socializing with friends who smoke), such a game environment may enable smokers to virtually practice and retain coping skills and build crucial self-efficacy skills for resisting smoking urges. In controlled pilot studies, virtually presented smoking cues have been shown to elicit more cravings than static photographs presented during traditional therapies [16-19]. Thus, a system that combines virtually presented tobacco cues with engaging narrative and personally relevant coping skills practice may help smokers overcome barriers to quitting and tobacco abstinence. Unlike virtual craving studies or existing game platforms, our project aims to develop a cessation treatment application using an immersive laptop or tablet-based game environment to help smokers cope with smoking urges and prevent smoking lapses.

### Design of QuitIT: Structure

We envision the game as a series of intertwining episodic stories [20], which link various characters with an ultimate goal. Ultimately, the player will be introduced to a group of 4 travelers stranded at an airport during a blizzard as they sit at a restaurant and relate stories of how they quit smoking. Then, the players will be taken to flashback scenarios in which they attempt to reenact the characters’ trigger situations without smoking. Each flashback will open up only after the previous one is resolved with the goal for the player of ensuring that the characters (“travelers”) get to tell their story of confronting smoking urges.

### Design of QuitIT: Behavioral Theory

**Figure 1:**
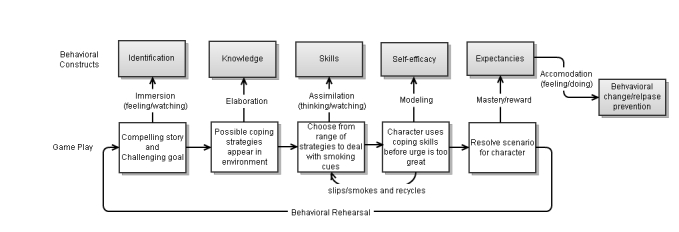
Integration of game mechanics with behavior change theory.

We developed the game play on the basis of principles from Social Cognitive theory (see Figure 1), which have been associated with tobacco cessation [21].

The game employs a narrative story-based structure to promote immersion in the game, emotional arousal, and a sense of direct experience and change in attitude toward tobacco cessation [22,23]. In each episode, the players select some of the character’s thoughts and dialogue, which increases narrative immersion. Game mechanic and parallel behavioral constructs are shown in Table 1.

Learning to monitor and manage urges to smoke is a core aspect of tobacco cessation treatment; therefore, an “urge meter” serves as the hook to engage decision-making and game play [24]. If a player fails to enact sufficient coping strategies their “urge meter” continues to increase until he or she “slips” and smokes followed by an opportunity to play the scene again to achieve mastery, which is a scenario similar to what the smokers would encounter in everyday life. Thus, our system has the core aspects of a serious game in that it presents a clear goal and rules for the player within the context of teaching behavior change [20].

**Table 1:** Game mechanics and related behavioral constructs.

Game mechanic	Construct
Story, narrative, and internal dialogue	Identification
Menu of coping strategies appears in game	Knowledge
Players must match optimal coping strategy to each smoking cue	Skills
Characters act out coping methods	Self-efficacy
Characters succeed at avoiding smoking and are rewarded	Expectancies

### Design of QuitIT: Prototype

To develop the initial prototype for testing, researchers from Memorial Sloan-Kettering Cancer Center (MSKCC) in New York City (Ostroff, Burkhalter, and Krebs) performed a series of in-person and remote meetings with the design team of Muzzy Lane (ML), a Boston-based game development company, to discuss methods for translating behavioral coping strategies into a game environment. The ML team then developed an initial game design approach consulting weekly with the MSKCC team and sharing files via a Web-based project management program to review screenwriting and design features. Our game design followed an iterative testing process generally recommended in software development, first creating and evaluating an alpha version of a functioning prototype [25]. Before creating a working prototype for alpha testing, we convened a patient advisory group of 4 volunteers from MSKCC’s patient volunteer program who had a history of smoking. The advisory group was presented with a non-electronic mock-up (using board-game metaphors) to allow the team to review the game concepts and play. Their feedback (1) reinforced the idea of using a narrative storytelling element and (2) suggested that the characters should be able to enact internal struggles as well as confront environmental cues to smoke. This final element was integrated into the game play.

**Figure 2:**
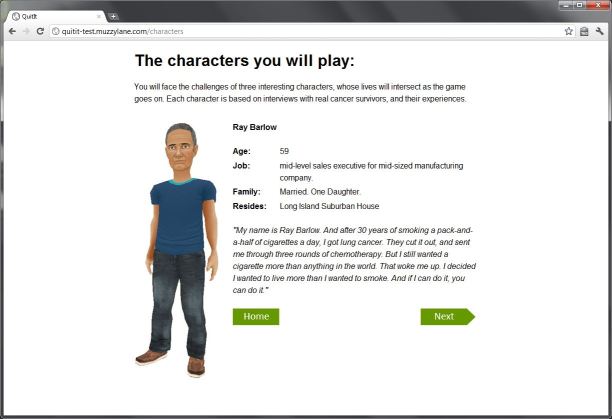
Introduction to Ray, the main character.

**Figure 3:**
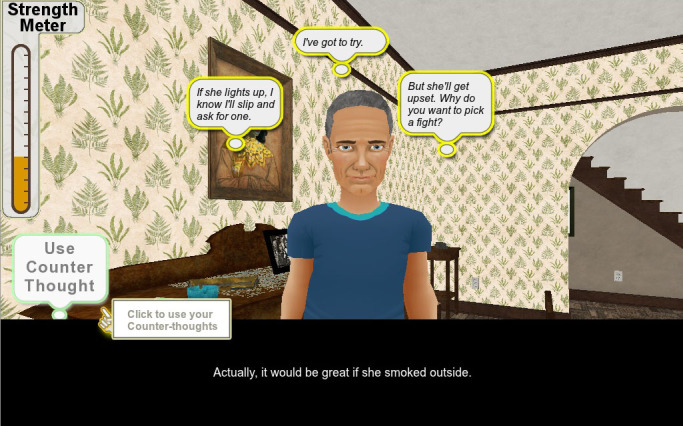
Players choose how Ray thinks through a cue situation.

**Figure 4:**
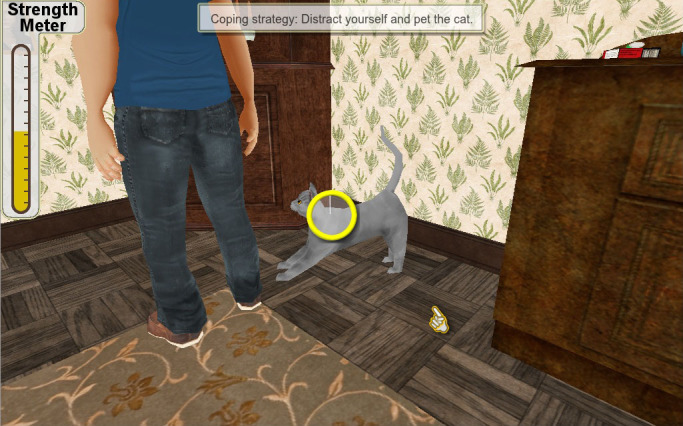
Example of using behavioral coping strategy.

Our alpha version was based on one trigger episode in which a character, Ray, struggles with trying not to smoke after dinner. The scene and dialogue between the character (Ray) and other virtual characters (his wife and teenage daughter) were scripted by a professional screenwriter and recorded in a sound studio (see Figures 2-4 for screenshots). This paper describes feedback from the alpha testing phase performed with tobacco cessation experts (n=5) and current and former smokers (n=20) with the aim of providing information useful for development of the health game.

## Methods

### Overview

Our evaluation methods were similar to those advocated by Moreno-Ger et al for assessing serious games and as applied in alpha testing protocols for health games [26,27]. We employed both expert and user methods, and the user methods consisted of dual observational and survey approaches.

### Expert Panel Review

A 90-minute semi-structured interview was conducted to introduce the game and solicit feedback on the prototype from 5 external consultants with expertise in the development and evaluation of tobacco cessation interventions. These consultants were provided with off-site access to the prototype game and were requested to provide specific feedback on the relevance, usability, and utility of the game. The group interview was digitally recorded for content analysis.

### Participant Recruitment

We recruited 20 adults who were former and current smokers treated by the MSKCC Tobacco Cessation Program to serve as game testers. Participants were contacted and screened for interest by phone. Eligible participants were individuals over 30 years of age, with a history of cancer and smoking, and with sufficient dexterity and vision to manipulate a computer and mouse. Interested smokers were scheduled to evaluate the game at the MSKCC Communication Skills Training Laboratory, a facility with large-screen monitors and digital video recording devices. Once the participants provided informed consent, Dr. Krebs conducted the patient feedback sessions. A $25 cash incentive for participation/travel reimbursement was provided. The study was approved by the MSKCC Institutional Review Board.

### End-User Testing

To gather user feedback on the prototype, we followed a mixed methods protocol (ie, structured questions and “talk aloud”), which is recommended for usability testing [26]. Game testers were given a brief introduction about the use of the game followed by 30 minutes of free-form game play. Testers used the same iteration of the game as that used by the expert panel. Because the ultimate goal is for the game to be self-directed, only minimal in-person instruction was provided. Testers were encouraged to verbalize their thoughts and raise questions as they played through the prototype of an after-dinner scene with the character “Ray” and his family. After exploring the scene, the testers were asked a series of open- and closed-ended Likert-type questions that assessed the game interface, relevance of tobacco cessation content, overall experience, and cultural appropriateness, as suggested for the evaluation of health communication programs [28]. In addition, usability was assessed using the 10-item System Usability Scale (SUS). The wording was slightly modified to represent “game” instead of “system.” The SUS is scored on a scale of 1-100 [29]. Testing sessions were video-recorded showing the screen and user for subsequent thematic analysis.

### Analysis

Data sources consisted of audio/video recordings of user testing sessions, session notes, and closed and open-ended responses to the assessment. Descriptive statistics were calculated for quantitative measures. Drs. Krebs and Ostroff reviewed recordings, open-ended responses, and session notes and identified key themes according to the standards of analysis for qualitative data [30]. Themes were discussed and finalized through a consensus process.

## Results

### Expert Panel Feedback

The Expert Panel provided the three primary themes and suggestions as follows:

1. *A clear and compelling initial orientation about the goal of game play and explicit instructions for using the game environment are required*. Experts recognized that our intended users are not experienced game players and therefore suggested that greater attention be paid to “setting the stage” and framing the game. Similarly, the experts suggested that less experienced game players might find it difficult to engage the interactive elements of the game environment and suggested that a narrator and/or “help” icon could help players more easily navigate the game environment. Although they liked the overall concept of selecting “Counter Thoughts” as coping strategies, the experts felt that either a demonstration or explicit coaching from a narrator would be required to reduce confusion in distinguishing between the bubbles showing “Counter Thoughts” versus those for Internal Dialog.
2. *The game characters should exemplify the demographics of a broad range of users.* Experts discussed the pros and cons of having users play themselves or a game character. One expert suggested that being able to build, select, and personalize the character is a fun way to increase the players’ game engagement. All agreed that having choices with regard to character selection and options for play will enhance relevance.
3. *Make the game more engaging by amplifying feedback to the user for helpful versus unhelpful game decisions*. Several comments focused on the form and functionality of the “urge meter.” The experts found the changing color (green, yellow, orange, red) to be too subtle and suggested that we add “happy” or “loser” sounds depending upon whether the player made a game decision that raised or lowered the urge meter (“the meter should be more lively”). Two of the experts suggested that some sort of familiar gauge (speedometer, gas tank [E/F] meter) would provide more compelling visual feedback. One expert suggested adding more explicit praise and encouragement for constructive use of coping strategies. Another suggested that effective use of coping strategies be reinforced with evidence of the character having powered-up (acquired some wisdom or mastery of coping strategy) or with a summary debriefing statement what the player has learned in prior game play.

### End-User Characteristics

The demographic characteristics of the current or former smokers (n=20) from the MSKCC Tobacco Cessation Program who were recruited as volunteers for testing are shown in Table 2. Participants spanned a wide range of ages from 31 to 74 years, with a mean age of 56 years. Participants were mostly women (14/20, 70%), with a high representation (40%) of racial/ethnic minorities. Current smokers comprised 65% (13/20) of the sample. Regarding computer and gaming experience, 30% (6/20) did not use a computer even occasionally and 80% (16/20) had little or no prior experience of games played on a computer or game console. We did not assess the use of other game modalities (eg, mobile apps).

### End-User Feedback

Items evaluated four important domains as follows: User Interface, Usability, Content, and Overall Experience. User interface, defined by the ability to figure out how to play the game, understand the instructions and text, know what a user is supposed to do, comfort in playing the game, and professionalism was rated at a moderate to high level using a 5-point Likert scale ranging from 3.00 to 4.65 (see Tables 2 and 3). Compared to other commercial computer systems, the SUS (1-100) summary score was in the average range (mean 67.00) (Table 3) [29]. The content items assessed the game’s utility in helping users manage smoking urges (mean 2.90), prevent relapse (mean 3.65), and apply the content to their own lives (Table 4). Testers rated content relevance from a moderate to a high level (mean 4.10). In terms of their game experience, testers reported moderate to high satisfaction (mean 3.75), would strongly recommend it to others (mean 4.70), and felt that the game kept their attention (mean 3.50).

Finally, responses from open-ended questions and patient comments were transcribed and thematically coded. Six primary themes emerged from the qualitative feedback:

1. *The user interface was challenging until some instructions regarding game play were provided.* Testers described that it was easy to play “after initial guidance” and that the “meter going up meant I was doing well.” On the other hand, testers said “Instructions needed to be more explicit.” It became clear that our completed version will require the game to begin with a demonstration and orientation to the game features. The simple clickable user interface succeeded in making the game accessible in that as soon as testers were given a brief introduction, even players who had never used a computer before were able to use it easily.
2. *Testers strongly identified with the smoking-related trigger situations and Ray’s (the primary character) struggle to remain smoke-free.* When asked what they liked most about the game, testers strongly affirmed the authenticity of the after dinner scene in which they played the character, Ray. For instance, testers said “I knew what he was going through. I related to the situations,” and “I related to Ray; I was feeling everything he was feeling.” Testing revealed that users strongly identified with the character and dialogue of the situation. No user stated that he or she would rather have played a character representing him or herself. Testers responded that: “I found myself projecting a lot” and “The thought choices were spot on with what you’d do or say to yourself.”
3. *The process of game play demonstrated both behavioral and cognitive coping skills for remaining smoke-free*. Testers responded that the game play was useful for teaching and reinforcing coping skills: “I like that I was brought along as the character, since it introduced me to new ideas about how to not smoke,” and “[The game] shows you how not to escalate situations and make things worse.”
4. *Testers made suggestions for broadening the characters’ experience, adding coping situations, and for reflecting their own experiences with cancer.* “He needs to be able to do more things.” Testers suggested puzzles, reading, going outside, exercising, doing artwork, praying, and clearing dishes. They noted that it should be “More in-depth about harsh realities [of smoking].” Testers expressed desire for a “Female character in a management job”, that the game needed a “dark-skinned character” and that we should “Add more races and realistic situations for those races.”
5. *Testers noted suggestions for making the game more fun and fast-paced.* While testers found the game interesting and engaging, they also expressed a desire for it to have more elements of fun. They stated that we “Need to perk it up,” and that Ray was a “glum character.” In line with typical expectations of a game, testers also wanted a reward structure: “I wanted a reward. I wanted it to keep score in the end”, and that “Winning reinforces positive coping.” They thought that the action should move along more quickly as well and found that “All the choices slowed you down” and “took too long to read.”
6. *The game offers strong potential to be useful for preventing smoking lapses.* In their summary comments, testers remarked that the game “Reinforced tools and strategies I’ve learned,” that it would “Help me in situations where I have a pattern and need to see it differently,” and that “Because I'm slipping, it’s a good reminder, feels motivating.” Participants also liked the computer model in that “Interactive is the way people are going to be taught,” that the “Computer idea is good because people can relate to it,” and “I was fascinated because I’ve never seen anything like it.” One patient noted that “It raised my awareness more so than ads on TV” in reference to graphic anti-smoking ads appearing in the New York City area.

**Table 2:** Phase I prototype evaluation: participant characteristics (n=20).

Participant characteristics		n (%)
**Sex**		
	Male	6 (30%)
	Female	14 (70%)
**Race/ethnicity**		
	African-American	7 (35%)
	Hispanic	1 (5%)
	Non-Hispanic White	12 (60%)
**Education**		
	High school or less	4 (20%)
	Some college/degree	6 (30%)
	Graduate/professional	10 (50%)
**Smoking status**		
	Current	13 (65%)
	Former	7 (35%)
**Use a computer at least occasionally?**		
	Yes	14 (70%)
	No	6 (30%)
**Use a game console?**		
	Seldom/never	16 (80%)
	Every few days	2 (10%)
	Once or more a day	2 (10%)

**Table 3:** Phase I prototype evaluation: interface and usability results (n=20).

Game evaluation		Scale	n (%)	Mean (SD)
**User interface**				
	How easy or difficult was it to figure out how to play?	1=Very difficult	0 (0)	3.55 (0.89)
		2=Difficult	2 (10)	
		3=Neutral	8 (40)	
		4=Easy	7 (35)	
		5=Very easy	3 (15)	
	How easy or difficult was it to understand the instructions?	1=Very difficult	1 (5)	3.70 (1.13)
		2=Difficult	2 (10)	
		3=Neutral	4 (20)	
		4=Easy	8 (40)	
		5=Very easy	5 (25)	
	How easy or difficult was it to see the on-screen text?	1=Very difficult	0 (0)	4.65 (0.59)
		2=Difficult	0 (0)	
		3=Neutral	1 (5)	
		4=Easy	5 (25)	
		5=Very easy	14 (70)	
	How easy or difficult was it to know what you were supposed to do?	1=Very difficult	2 (10)	3.00 (1.08)
		2=Difficult	4 (20)	
		3=Neutral	7 (35)	
		4=Easy	6 (30)	
		5=Very easy	1 (5)	
	How easy or difficult was it to know whether you were doing well?	1=Very difficult	1 (5)	3.65 (1.04)
		2=Difficult	1 (5)	
		3=Neutral	6 (30)	
		4=Easy	8 (40)	
		5=Very easy	4 (20)	
	How comfortable or frustrated did you feel interacting with the game?	1=Very frustrating	0 (0)	3.85 (1.14)
		2= Frustrating	4 (20)	
		3=Neutral	2 (10)	
		4= Comfortable	7 (35)	
		5=Very Comfortable	7 (35)	
	How would you rate the professionalism or production value of the game?	1= Poor	2 (10)	3.95 (1.23)
		2=Somewhat poor	0 (0)	
		3=Moderate	3 (15)	
		4=Good	7 (35)	
		5=Very good	8 (40)	
**Usability**				
	Summary score	0-25	0 (0)	67.00 (14.81)
		26-50	2 (10)	
		51-75	12 (60)	
		76-100	6 (30)	

**Table 4:** Phase I prototype evaluation: cessation content and overall rating (n=20).

Evaluation questions			Scale	n (%)	Mean (SD)
**Smoking cessation content**					
		How much did you learn about ways to manage smoking urges?	1=Not much at all	2 (10)	2.90 (0.91)
			2=Learned some things	3 (15)	
			3=Learned a little bit	10 (50)	
			4=Learned a great deal	5 (25)	
		Would you use this game to prevent smoking relapse in the future?	1=Definitely not	1 (5)	3.65 (1.04)
			2=Probably not	3 (15)	
			3=Might or might not	4 (10)	
			4=Probably would	10 (50)	
			5=Definitely would	2 (10)	
		Will you be able to apply what you learned to your home environment?	1=Definitely not	0 (0)	4.10 (1.07)
			2=Probably not	3 (15)	
			3=Might or might not	1 (5)	
			4=Probably would	7 (35)	
			5=Definitely would	9 (45)	
**Overall experience**					
	Please rate your overall experience with the game		1=Extremely dissatisfied	1 (5)	3.75 (0.85)
			2=Dissatisfied	0 (0)	
			3=Neutral	4 (20)	
			4=Satisfied	13 (65)	
			5=Extremely satisfied	2 (10)	
	Would you recommend the game to others?		1=Definitely would not	0 (0)	4.50 (0.69)
			2=Probably would not	1 (0)	
			3=Might or might not	2 (10)	
			4=Probably would	6 (30)	
			5=Definitely would	12 (60)	
	To what extent did the game keep your attention?		1=Definitely not	0 (0)	3.50 (0.61)
			2=Not very much	1 (5)	
			3=Somewhat	8 (40)	
			4=Very much	11 (55)	

## Discussion

### Principal Results

The aim of the present study was to inform the development of a game for enhancing the skills required for coping with smoking urges. We employed a combination of qualitative and quantitative evaluation methods from content experts and end-users. Findings from the tests centered on (1) usability, (2) character identification, and (3) player engagement.

For improved usability, the experts and users suggested the need for an introduction with clearer instructions, a demonstration of game play, and a help menu available throughout game play. User ratings of the interface ranged from difficult to easy, with the poorest scores for “knowing what you were supposed to do” and “figuring out how to play.” A usability score of 67 for this prototype falls at the 49^th^ percentile compared to that obtained in other studies using the SUS scale [29]. Such usability results are in line with our qualitative data, which indicate that users and experts wanted more explicit instructions. This feedback is similar to that reported by a similar population of older cancer patients in evaluation of a treatment decision-making game [27]. In addition, patient testing showed problems with the design of the urge meter as many noted that they did not attend to it. During alpha testing of a dietary intervention game, Beltran et al [31] also found that testers wanted clear instructions and had difficulty attending to a similar feedback meter. More attention to setting up game play in the final version is warranted as clarity of goals and ease of use is critical for ensuring player engagement. These data indicate that the next iteration will need to improve the balance between story immersion and game mechanics.

Users identified with the characters, which indicated that the writing and script accurately reflected challenging situations smokers typically encounter. A player’s ability to identify with and interact as a game character is particularly important for narrative suspension of disbelief and is the key difference between a game-based intervention and one that focuses on more traditional health education approaches. Comments indicating that players found themselves “projecting” and “feeling everything he was feeling” indicate that the narrative engaged players’ emotions, which can be important for maintaining interest in playing, shifting attitudes toward behavior change, and promoting retention of learning [32,33]. Indeed, functional magnetic resonance imaging studies with the chemotherapy-related Re-Mission game have suggested that players take on the goals of the game characters, with the game activating brain centers related to reward and motivation [34]. The design team had lengthy discussions about the implications of having the players create characters representing themselves on engagement. It appears this option was not required as players readily identified as the characters with none stating they would rather have created their own avatars. On the other hand, both experts and users suggested adding characters and situations that reflect greater diversity across socio-economic and ethnic/racial backgrounds. It is interesting to note that testers who seemed to have less insight into their own smoking gave the game more positive reviews than others who could readily discuss quitting strategies. Thus, a game modality may be particularly suited to communicating and teaching cessation skills to those who benefit from experiential learning. This also suggests a challenge in designing a game that is equally interesting and helpful for smokers at various stages of change. It would need to employ and exemplify both cognitive and emotional processes most relevant in pre-Action stages as well as behavioral strategies to help those starting out with changes [35].

Third, the game held users’ attention and promoted expansion of learning about their own behaviors. Comments such as, “[it taught me] to be mindful of thoughts and [that you can] can stop yourself to pause and make a choice” and “I learned substitution, distraction, and avoiding cues” indicate that players took away new ideas for how to cope with urges to smoke. One player even observed that choosing assertive and conflict-diffusing dialogue decreased the urge meter, a subtle yet important insight gleaned from the game mechanics. This evidence of high-level learning is encouraging as the game itself did not take a didactic approach to these concepts - players arrived at these conclusions simply via the characters’ modeling within the game. On the other hand, users and expert reviewers wanted a more integrated scoring system and to improve the “fun” aspects of the game. This is important as perception of a game as enjoyable has been found to be positively correlated with self-efficacy and intentions for behavioral outcomes [36]. It is encouraging that users and experts clearly see the utility of the game, which is essential to achieving “buy-in” from both a health care provider and patient/client perspective.

### Limitations

The primary goal of this study was to gather feedback to inform further development of the game. Protocols specific to evaluating health games, such as those outlined by Moreno-Ger et al [26], were not yet available when we performed our testing. Nevertheless, we followed standard methods for developing health interventions [28], which were quite similar to those reported previously, and they yielded useful results. The number of individual interviews and testing sessions was adequate to achieve saturation of data [26,30]. One limitation of our study was use of the SUS, which as Moreno-Ger et al note is most applicable to assessing business software applications [26]. The items, however, primarily focus on ease of use and were helpful in quantifying that aspect of the game. The population was diverse in terms of age, race, and gender, but included only smokers with a history of cancer. While this is our target end-user population, it may limit the generalizability of the final game as applied to other populations.

### Comparison With Previous Studies

A multitude of smartphone apps and interactive “tailored” programs are available for smoking cessation [37-39], but these focus on straightforward education and behavioral management, what Winn [40] refers to as a “third-person symbolic” experience. On the other hand, health behavior change games represent a promising new application for games as they shift from an emphasis on objectivist representation of knowledge (ie, “smoking carries these risks”, “use this method to stop”) to a constructivist or problem-based approach. Problem-based learning engages the user on a first-person basis as one moves through an environment, encountering and working through situations. Such an engagement framework [10] facilitates key features of active learning, namely, defining a task, practicing skills, taking risks without negative consequences, and receiving immediate feedback, all of which increase motivation, which is key in effecting behavior change [22,23,41-43]. The immersion of a user into a game character’s narrative allows the user to code information in a “first-person nonsymbolic” experience and likely engages emotional arousal more effectively than other types of interventions [32]. The process of making decisions and skill practice in a realistic environment thus improves the potential for meaningful learning and maintenance of behavior change [22]. Our game’s focus on narrative structure and integration of a range of evidence-based strategies for tobacco cessation offer significant strength as an innovative behavioral intervention method.

### Future Directions

**Figure 5:**
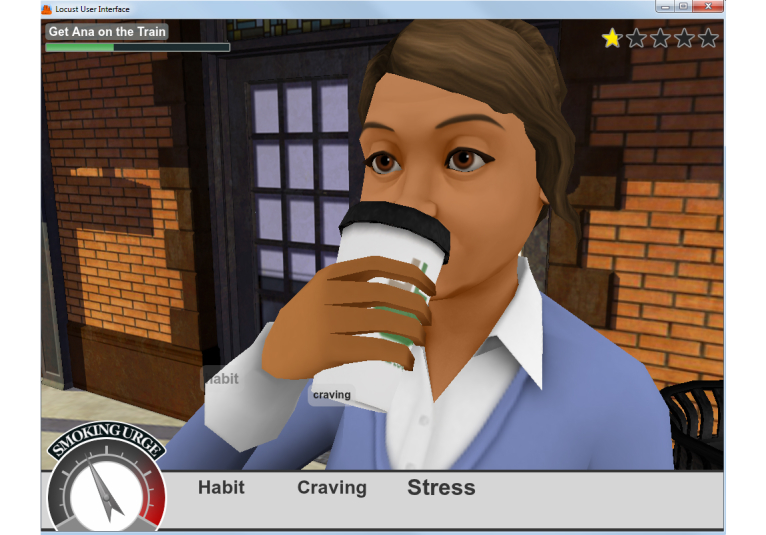
Example of revised game interface.

A large number of improvements to the game are being integrated into the next version as a result of the alpha testing feedback. We addressed a number of usability issues by replacing the thermometer-style “urge meter” with a large pressure gauge meter. The urge triggers encountered in the storylines now clearly affect the urge to smoke (urge needle) and the user interface displays the triggers that have the biggest impact on the urge needle (see Figure 5). This new interface clearly indicates to players when and why they are in danger of a slip-up. Our prototype had several different modes for coping that proved confusing, such as searching the environment for coping tools. This has been streamlined so all coping tools, strategies, or items are presented to the player as a menu with between two to four choices. When a coping method is selected, there is immediate feedback; the urge needle goes down as appropriate, the strongest urge triggers re-arrange themselves if necessary, and a scoring system rewards the player for effective choices. A tutorial is included in the first episode, and all episodes conclude with an After Action Report to further illustrate the relationship between the effectiveness of particular strategies against certain situations. These improvements are designed to facilitate quick understanding of the game, which allows the player to focus on recognizing triggers and analyzing the coping methodologies that are effective against specific triggers. In addition, there has been a marked improvement in character animation, storytelling, and overall production values.

### Conclusions

Consistent with recent survey findings that smokers would be interested in using a video game for cessation [44], our results from the alpha prototyping phase show strong potential for a video game modality to enhance skills for coping with smoking urges. Video games are increasingly popular; indeed, “strategy” games, the genre under which our game falls, was the most popular computer game genre in 2011 comprising 27% of sales [12]. A coping skills game offers significant advantages to traditional behavioral treatment in that it can be practiced multiple times at smokers’ convenience to address their specific smoking triggers, can create realistic simulations that provide behavioral rehearsal opportunities that are not possible in real-world treatment settings (eg, social smoking situations), and can be readily disseminated to a broad audience of tobacco-dependent persons over a secure connection without resource-intensive one-on-one programs. In the next phase of game development, we will conduct a randomized clinical trial (Best Practices + Smoking Cues Coping Skills Game vs Best Practices Only) to test whether the game increases coping self-efficacy and smoking abstinence among hospitalized cancer patients.

## Abbreviations

ML: muzzy lane
MSKCC: Memorial Sloan-Kettering Cancer Center
SUS: System Usability Scale

## References

[1] Brummett BH, Babyak MA, Mark DC, Williams RB, Siegler IC, Clapp-Channing N, Barefoot JC (2002). Predictors of smoking cessation in patients with a diagnosis of coronary artery disease. J Cardiopulm Rehabil.

[2] Perez GH, Nicolau JC, Romano BW, Laranjeira R (2008). Depression: a predictor of smoking relapse in a 6-month follow-up after hospitalization for acute coronary syndrome. Eur J Cardiovasc Prev Rehabil.

[3] Wolfenden L, Campbell E, Walsh R, Wiggers J (2003). Smoking cessation interventions for in-patients: a selective review with recommendations for hospital-based health professionals. Drug Alcohol Rev.

[4] Brandon TH, Vidrine JI, Litvin EB (2007). Relapse and relapse prevention. Annu Rev Clin Psychol.

[5] Fiore M, Jaen, CR, Baker, TB (2008). Treating Tobacco Use and Dependence.

[6] Marlatt GA, Gordon JR (1985). Relaspe prevention: Maintenance strategies in the treatment of addictive behaviors.

[7] Sheffer CE, Stitzer M, Brandon T, Bursac Z (2010). Effectiveness of adding relapse prevention materials to telephone counseling. J Subst Abuse Treat.

[8] Irvin JE, Bowers CA, Dunn ME, Wang MC (1999). Efficacy of relapse prevention: a meta-analytic review. J Consult Clin Psychol.

[9] deFreitas S (2006). London: Joint Information Systems Committee.

[10] Dickey MD (2005). Engaging By Design: How Engagement Strategies in Popular Computer and Video Games Can Inform Instructional Design. Educational Technology Research & Development.

[11] Squire K (2005). MASIE Center.

[12] (2012). Entertainment Software Association.

[13] Primack BA, Carroll MV, McNamara M, Klem ML, King B (2012). Role of video games in improving health-related outcomes: a systematic review. Am J Prev Med.

[14] Lieberman DA (2001). Management of chronic pediatric diseases with interactive health games: theory and research findings. J Ambul Care Manage.

[15] Abroms LC, Padmanabhan N, Thaweethai L, Phillips T (2011). iPhone apps for smoking cessation: a content analysis. Am J Prev Med.

[16] Baumann SB, Sayette MA (2006). Smoking cues in a virtual world provoke craving in cigarette smokers. Psychol Addict Behav.

[17] Bordnick PS, Graap KM, Copp H, Brooks J, Ferrer M, Logue B (2004). Utilizing virtual reality to standardize nicotine craving research: a pilot study. Addict Behav.

[18] Kuntze MF, Stoermer R, Mager R, Roessler A, Mueller-Spahn F, Bullinger AH (2001). Immersive virtual environments in cue exposure. Cyberpsychol Behav.

[19] Lee JH, Ku J, Kim K, Kim B, Kim IY, Yang BH, Kim SH, Wiederhold BK, Wiederhold MD, Park DW, Lim Y, Kim SI (2003). Experimental application of virtual reality for nicotine craving through cue exposure. Cyberpsychol Behav.

[20] Baranowski T, Buday R, Thompson DI, Baranowski J (2008). Playing for real: video games and stories for health-related behavior change. Am J Prev Med.

[21] Gwaltney CJ, Metrik J, Kahler CW, Shiffman S (2009). Self-efficacy and smoking cessation: a meta-analysis. Psychol Addict Behav.

[22] Lu AS, Thompson D, Baranowski J, Buday R, Baranowski T (2012). Story Immersion in a Health Videogame for Childhood Obesity Prevention. Games for Health Journal.

[23] Hinyard LJ, Kreuter MW (2007). Using narrative communication as a tool for health behavior change: a conceptual, theoretical, and empirical overview. Health Educ Behav.

[24] Howland GH (2002). Balancing gameplay hooks. Laramée F. editor. Game design perspectives.

[25] Pagulayan RJ, Keeker K, Wixon D, Romero RL, Fuller T, Jacko JA, A S. (2003). User-centered design in games. The Human-Computer Interaction Handbook: Fundamentals, Evolving Technologies and Emerging Applications.

[26] Moreno-Ger P, Torrente J, Hsieh YG, Lester WT (2012). Usability testing for serious games: Making informed design decisions with user data. Advances in Human-Computer Interaction.

[27] Reichlin L, Mani N, McArthur K, Harris AM, Rajan N, Dacso CC (2011). Assessing the acceptability and usability of an interactive serious game in aiding treatment decisions for patients with localized prostate cancer. J Med Internet Res.

[28] U.S. Department of Health and Human Services (2002). Making Health Communication Programs Work.

[29] Sauro J (2011). A Practical Guide to the System Usability Scale.

[30] Silverman D (2006). Interpreting Qualitative Data. 3rd ed.

[31] Beltran A, O'Connor T, Hughes S, Baranowski J, Nicklas TA, Thompson D (2012). Alpha Test of a Videogame to Increase Children's Vegetable Consumption. Games for Health Journal.

[32] Lu AS, Baranowski T, Thompson D, Buday R (2012). Story Immersion of Videogames for Youth Health Promotion: A Review of Literature. Games for Health Journal.

[33] Baranowski T, Baranowski J, Thompson D, Buday R (2011). Behavioral science in video games for children's diet and physical activity change: key research needs. J Diabetes Sci Technol.

[34] Cole SW, Yoo DJ, Knutson B (2012). Interactivity and reward-related neural activation during a serious videogame. PLoS One.

[35] Sun X, Prochaska JO, Velicer WF, Laforge RG (2007). Transtheoretical principles and processes for quitting smoking: a 24-month comparison of a representative sample of quitters, relapsers, and non-quitters. Addict Behav.

[36] Peng W (2009). Design and evaluation of a computer game to promote a healthy diet for young adults. Health Commun.

[37] Elfeddali I, Bolman C, Candel MJ, Wiers RW, de Vries H (2012). Preventing smoking relapse via Web-based computer-tailored feedback: a randomized controlled trial. J Med Internet Res.

[38] Krebs P, Prochaska JO, Rossi JS (2010). A meta-analysis of computer-tailored interventions for health behavior change. Prev Med.

[39] Riley WT (2012). Leveraging technology for multiple risk factor interventions. Arch Intern Med.

[40] Winn W (1993). A conceptual basis for educational applications of virtual reality.

[41] Bowman R (1982). A 'Pac-man' theory of motivation: Tactile implications for classroom instruction. Educational Technology.

[42] Malone TW (1981). Toward a Theory of Intrinsically Motivating Instruction. Cognitive Sci.

[43] Provenzo E (1991). Making Sense of Nintendo.

[44] Raiff BR, Jarvis BP, Rapoza D (2012). Prevalence of video game use, cigarette smoking, and acceptability of a video game-based smoking cessation intervention among online adults. Nicotine Tob Res.

